# Purkinje trigger mapping in postinfarction ventricular fibrillation storm with ventricular abnormal activities detection

**DOI:** 10.1016/j.hrcr.2025.10.021

**Published:** 2025-10-22

**Authors:** Hiroyuki Kono, Kenichi Hiroshima, Michio Nagashima, Masato Fukunaga, Kengo Korai, Kenji Ando

**Affiliations:** Department of Cardiology, Kokura Memorial Hospital, Kitakyushu, Japan

**Keywords:** Ventricular fibrillation, Mapping, Catheter ablation, Purkinje denetworking, Premature ventricular contraction

**Key Teaching Points**

• Purkinje triggers in postinfarction ventricular fibrillation (VF): VF storms in the acute phase after myocardial infarction are often initiated by premature ventricular contractions (PVCs) arising from surviving Purkinje fibers in the infarct border zone. Recognizing these Purkinje-related PVCs as critical triggers is essential, especially when conventional antiarrhythmic therapy fails.
• Advanced mapping to identify slow conduction: the CARTO electroanatomic mapping system’s early meets late (EML) function can highlight zones of slow conduction and abnormal potentials, such as delayed Purkinje potentials or local abnormal ventricular activities, within scarred myocardium. Adjusting EML timing thresholds to create ventricular abnormal activities detection maps during sinus rhythm can help pinpoint conduction channels that may harbor VF triggers and drivers.
• Purkinje denetworking as rescue therapy: catheter ablation targeting the Purkinje network—known as Purkinje denetworking—is an emerging rescue therapy for refractory VF storm. Systematic ablation of Purkinje potentials within the infarct zone can eliminate both initiating PVCs and the sustaining substrate, often rendering VF noninducible. Mechanical circulatory support is invaluable to stabilize patients during such complex procedures in hemodynamically unstable VF storms.

## Introduction

Electrical storm, defined as 3 or more episodes of sustained ventricular arrhythmia within 24 hours, is a life-threatening condition. Post–myocardial infarction (MI) ventricular fibrillation (VF) storms are often triggered by premature ventricular contractions (PVCs) originating from surviving Purkinje fibers within the infarct border zone.^1^ In the acute or subacute MI phase, ischemic injury to the Purkinje system may cause abnormal automaticity or reentry, whereby a PVC precipitates VF.^2^^,^^3^

When antiarrhythmic drugs and defibrillation fail to control Purkinje-mediated VF storm, urgent catheter ablation should be considered. In a multicenter study, ablation of Purkinje-related PVCs terminated VF storm in 84% of post-MI patients, with each day’s delay in ablation increasing in-hospital mortality by approximately 11%.^4^, ^5^, ^6^

We previously reported the use of ventricular abnormal activities detection (VAAD) mapping—created with the CARTO early meets late (EML) function—to identify the critical isthmus of ventricular tachycardia (VT).^7^ To the best of our knowledge, this is the first report of acute-phase post-MI VF storm in which VAAD mapping guided a Purkinje denetworking (PDN) strategy, resulting in durable arrhythmia suppression.

## Case report

A 66-year-old man presented with acute, extensive anterior wall MI. Transthoracic echocardiography showed 25 % of left ventricular ejection fraction and diffuse hypokinesis. Coronary angiography revealed complete occlusion of the proximal left anterior descending artery (segment #6) and 99% stenosis of the mid-right coronary artery (segment #2). Both lesions were treated with percutaneous coronary intervention, and the early post–percutaneous coronary intervention course was uneventful for 3 days.

**Figure 1:**
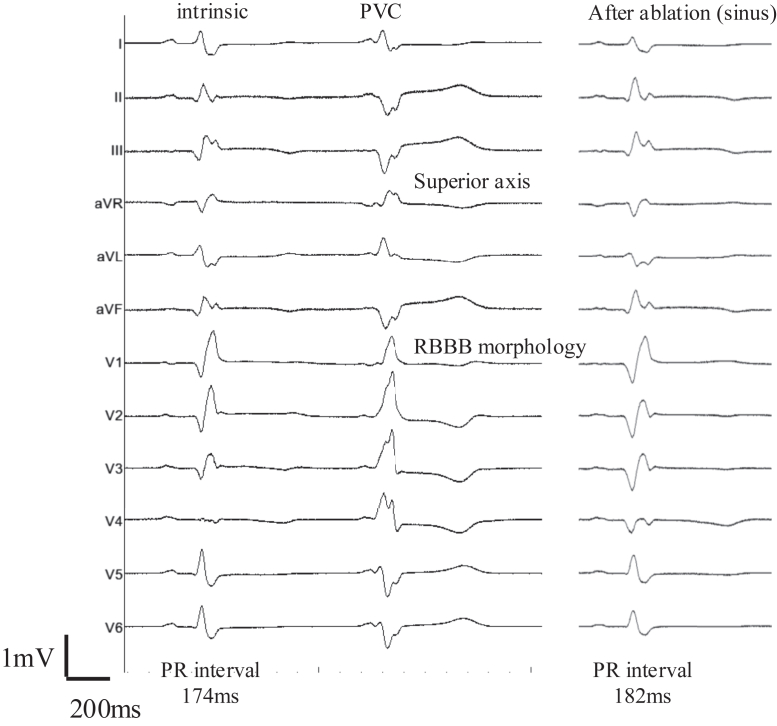
12-lead electrocardiogram during sinus rhythm showing the culprit PVC that consistently triggered VF. The PVC exhibits a complete right bundle branch block morphology and superior axis deviation (*red arrows*), consistent with an origin from the inferior septal left ventricular Purkinje network. After ablation, neither the PR interval nor the QRS axis changed. Calibration: 1 mV vertical and 200 ms horizontal. PVC = premature ventricular contraction.

On hospital day 4, he developed a VF storm, requiring multiple defibrillations. Each VF episode was consistently preceded by frequent PVCs with right bundle branch block (RBBB) morphology and superior axis on the 12-lead electrocardiogram (Figure 1), suggesting a left ventricular Purkinje origin from the inferior septum. During sinus rhythm, the electrocardiogram showed complete RBBB and left posterior fascicular block. Despite intravenous amiodarone and lidocaine, VF recurred. Rapid progression to cardiogenic shock necessitated venoarterial extracorporeal membrane oxygenation (VA-ECMO) and Impella support.

**Figure 2:**
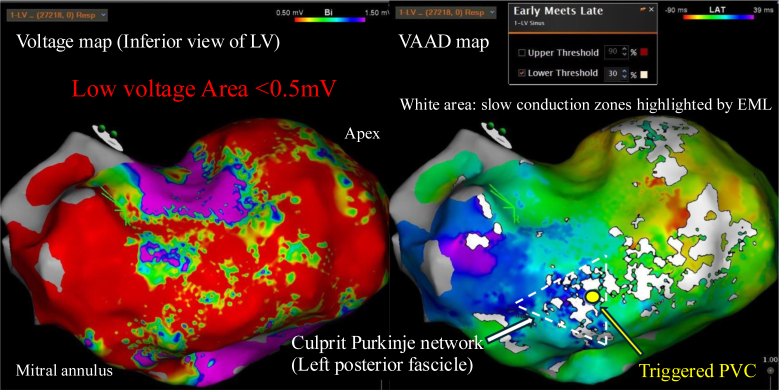
Electroanatomic maps of the left ventricle in sinus rhythm. *Left:* Voltage map illustrating an extensive low-voltage scar (<0.5 mV) from the inferior wall to the apex. *Right:* VAAD map created with the EML algorithm at a 30% lower threshold, revealing slow conduction zones (*white*) within the scar. The culprit Purkinje network (*white arrow*) and the earliest activation site of the triggered PVC (*yellow arrow*) are highlighted. The color bar indicates LAT in ms. EML = early meets late; LAT = local activation time; LV = left ventricular; PVC = premature ventricular contraction; VAAD = ventricular abnormal activities detection.

Because of refractory VF storm, urgent catheter ablation was performed on hospital day 6 under general anesthesia with full circulatory support. 3-dimensional electroanatomic mapping (CARTO 3, Biosense Webster, Diamond Bar, CA) during sinus rhythm revealed an extensive low-voltage scar from the inferior wall to the apex. Voltage mapping alone could not localize abnormal signals, so VAAD mapping was created using the EML function. The EML lower threshold was initially varied from 10% to 30% of the total activation time. A setting of 30% provided the clearest delineation of slow conduction zones with consistent correlation to discrete Purkinje potentials, while minimizing noise that was more evident at lower thresholds (Figure 2). Along these zones, discrete, high-frequency Purkinje potentials preceded the local ventricular electrogram.

**Figure 3:**
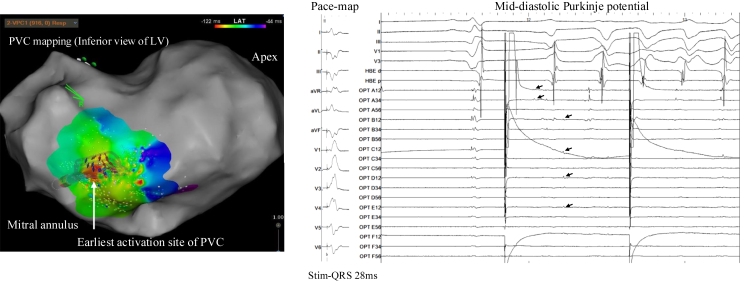
*Left:* Activation map during the culprit PVC showing the earliest activation site (*white circle*) in the inferoseptal LV, colocalized with Purkinje potentials. *Right:* Intracardiac electrograms from this site demonstrate a short stimulus-to-QRS latency (28 ms) during pace mapping, reproducing the clinical PVC morphology, and mid-diastolic Purkinje potentials (arrows) immediately preceding VF onset. The subsequent pacing shown after VF onset represents programmed pace map pacing that continued inadvertently because VF occurred unexpectedly during the pacing protocol. These findings implicate the Purkinje network in both triggering and maintaining VF. LV = left ventricular; PVC = premature ventricular contraction; VF = ventricular fibrillation.

High-density activation mapping of the frequent PVCs (Optrell multipolar catheter, Biosense Webster, Diamond Bar, CA) identified the earliest activation at the inferoseptal left ventricle, colocalized with the Purkinje potentials. Pace mapping produced an identical QRS morphology with a short stimulus-to-QRS latency (28 ms). During VF initiation, mid-diastolic Purkinje potentials were observed just before VF fragmentation (Figure 3), implicating the Purkinje network in both triggering and maintaining VF.

**Figure 4:**
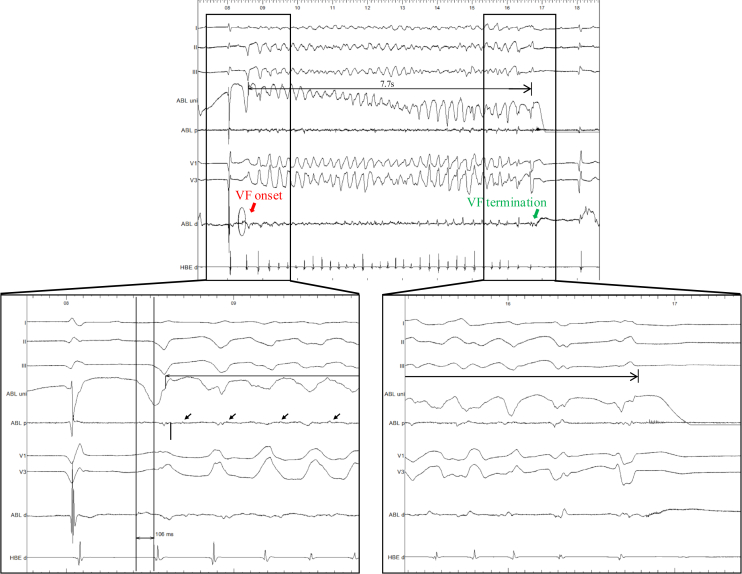
Intraprocedural electrogram recordings during ablation at the PVC origin. Radiofrequency delivery induced VF (*red arrow*), which spontaneously terminated after 7.7 seconds (*green arrow*) without external defibrillation. *Lower left:* Zoomed-in tracing shows mid-diastolic Purkinje potentials (*arrows*) recorded at the ablation site during VF. *Lower right:* Subsequent zoomed-in view demonstrates disappearance of these Purkinje potentials during ablation, coinciding with VF termination. After this, linear lesions were applied to complete Purkinje denetworking, after which VF was no longer inducible. VF = ventricular fibrillation.

Radiofrequency (RF) ablation (QDOT Micro, Biosense Webster) was first delivered at the PVC origin, promptly eliminating the trigger. After initial focal ablation targeting the earliest PVC site, VF was reinduced during RF delivery and terminated spontaneously within 7.7 seconds (Figure 4). Although spontaneous termination could theoretically occur, all previous VF episodes in this patient had required external defibrillation. This termination coincided with the disappearance of local Purkinje potentials, suggesting that ablation-induced network disruption contributed to arrhythmia cessation. In addition, VAAD mapping revealed a linear distribution of Purkinje potentials along the infarct border zone, indicating potential for multiple triggers. These findings, together with the acute-phase MI context, prompted a comprehensive PDN approach rather than focal ablation alone. Linear overlapping lesions were delivered along the infarct border zone and left posterior fascicle to disconnect Purkinje–myocardial junctions. After ablation, PVCs were absent, and VF was noninducible despite aggressive programmed stimulation on and off mechanical support. After ablation at the proximal sites, distal Purkinje potentials within the diseased area were no longer observed, supporting effective network interruption. Given that this was the first ablation session, performed in the setting of previous MI, and VT became noninducible, an epicardial approach was not pursued. VA-ECMO and Impella were removed within 24 hours. No ventricular arrhythmias recurred during hospitalization. A wearable cardioverter-defibrillator was prescribed for 3 months.

3 months after the MI, cardiac magnetic resonance imaging demonstrated late gadolinium enhancement in the region of the left posterior fascicle. Positron emission tomography–computed tomography performed at the same time revealed active myocardial inflammation localized to the same area, establishing a diagnosis of cardiac sarcoidosis. No extracardiac involvement was detected. For primary prevention, an implantable cardioverter-defibrillator was implanted, and oral prednisolone therapy was initiated.

At 1.5-year follow-up, no VT/VF recurrence or implantable cardioverter-defibrillator therapy has occurred. At follow-up, the left ventricular ejection fraction improved to 35%.

## Discussion

This case illustrates a rare but critical scenario: an acute-phase post-MI VF storm in a patient with cardiogenic shock, successfully managed by PDN guided by VAAD mapping. VF storms in the acute post-MI phase are frequently Purkinje mediated, given that surviving Purkinje fibers within the infarct border zone may develop abnormal automaticity or reentry.^8^, ^9^, ^10^, ^11^ The morphology of the PVC triggering VF—RBBB with superior axis—was highly suggestive of an inferior septal Purkinje origin.

### Technical aspect: VAAD mapping

Identifying critical Purkinje potentials within an extensive infarct scar is often difficult using voltage mapping alone. In this case, VAAD mapping with the CARTO EML function enabled functional substrate delineation during sinus rhythm by highlighting zones of slow conduction at a 30% activation time threshold. This threshold was selected after intraprocedural comparison of multiple settings, given that it provided the clearest visualization with minimal noise. The identified zones corresponded to discrete Purkinje potentials located along the infarct border, thereby facilitating precise localization of the arrhythmogenic substrate. This approach is conceptually aligned with other functional mapping strategies, such as isochronal late activation mapping and ripple mapping.^12^, ^13^, ^14^ Importantly, when different mapping methods were applied in combination, the location of the critical isthmus was consistently identified across modalities.^15^ Furthermore, VAAD mapping proved useful for detecting abnormal electrograms within the region of interest, and by lowering the EML threshold in the presence of residual inducibility, additional abnormal signals could be efficiently uncovered.

### Strategic aspect: PDN

After initial focal ablation, VF was reinduced and terminated spontaneously during RF application, coinciding with loss of Purkinje potentials. Together with the linear distribution of Purkinje signals along the scar border, these findings indicated broader Purkinje network involvement. Given the acute-phase MI setting and the risk of multiple triggers, we adopted a PDN strategy rather than focal ablation alone. Previous studies have reported high acute success rates with PDN in refractory VF,^16^, ^17^, ^18^, ^19^ albeit with a risk of conduction disturbances such as complete left bundle branch block.^18^ In our patient, PDN targeting the left posterior fascicle and adjacent Purkinje-rich border zones eliminated the trigger and rendered VF noninducible without new conduction block. Although activation mapping localized the PVC origin, it did not delineate the broader Purkinje network involvement. VAAD mapping, by contrast, revealed a linear distribution of Purkinje potentials along the infarct border, highlighting multiple zones of slow conduction beyond the focal PVC site. This incremental value over activation mapping was critical in guiding a denetworking strategy rather than focal ablation alone.

### Timing and support

The acute success in this case—termination of the storm and VF noninducibility—aligns with findings from Komatsu et al,^4^ who reported cessation of VF storm in 84% of post-MI patients after Purkinje trigger ablation, with each day’s delay in ablation increasing in-hospital mortality by approximately 11%. These data underscore the importance of early referral to a center capable of performing complex ablation in drug-refractory VF storm. In hemodynamically unstable patients, temporary mechanical circulatory support, as used here with VA-ECMO and Impella, can maintain organ perfusion and provide the procedural stability needed for high-resolution mapping and effective substrate modification.^20^

### Cardiac sarcoidosis

An additional unique feature of this case is the subsequent diagnosis of cardiac sarcoidosis. Similar to previous reports in which cardiac sarcoidosis was initially concealed by an acute ST-segment elevation MI presentation,^21^ the inflammatory process in our patient may have contributed to conduction system injury and arrhythmogenic substrate formation in conjunction with the infarct scar. The diagnosis was established according to the Japanese Circulation Society criteria, based on late gadolinium enhancement on cardiac magnetic resonance imaging involving the basal interventricular septum and left posterior fascicle, focal fluorodeoxyglucose uptake in the same area on positron emission tomography–computed tomography, and extracardiac findings consistent with sarcoidosis. Although post-MI inflammation can produce similar imaging findings, the distribution pattern involving the conduction system together with extracardiac involvement supported cardiac sarcoidosis as the more likely etiology. This case underscores the importance of considering infiltrative or inflammatory cardiomyopathy in the differential diagnosis of acute coronary syndromes, particularly when arrhythmia patterns suggest conduction system involvement.

### Limitations

This is a single case, limiting generalizability. Our institution has treated several post-MI VF storm cases with Purkinje-targeted ablation, but this is the first using VAAD-guided PDN. Larger prospective studies are needed to validate this strategy’s efficacy and safety.

## Conclusion

In acute-phase MI, Purkinje-targeted ablation guided by VAAD mapping can identify both the trigger and sustaining substrate of VF storm. PDN achieved durable suppression in this case. Early intervention with hemodynamic support should be considered for drug-refractory VF storm.

## Disclosures

The authors have no conflicts of interest to disclose.
